# Investment of a Dentist’s Earnings*Extract from address before the National Dental Association, Louisville, January 26, 1917, and reprinted from *Journal of N. D. A*.

**Published:** 1917-03

**Authors:** C. N. Johnson


### INVESTMENT OF A DENTIST'S EARNINGS.*

BY C. N. JOHNSON, M.A., D.D.S.

I believe that ordinarily speaking the first investment for a professional man should be in a home, then in life insurance to protect his family. These should be considered investments, not so much for profit as for protection, and this form of investment is more obligatory on a man than any other. I have heard the argument used frequently that it is cheaper to rent than to own your own home, but even if it can so be figured on paper it seldom holds true in fact. Usually the professional man who has accumulated anything worth while has at one time or another owned a home. Aside from the consideration of finance there are sentimental reasons why a professional man will do well to own a home. Home ownership anchors a man to a community in a more substantial way than anything else. He is a more responsible citizen with a keener interest in community affairs than if he is merely a tenant. As to life insurance it is frequently the sole barrier between one's family and want in case of a sudden taking away of the head of the home. And yet, I would not advise the form of insurance which compels a man to pay during his lifetime, and which is collectable only in case of death. This frequently works a hardship on a man in his last days at a time when the burdens of business should be eased. An endowment policy which matures in a given number of years, and can be realized on at a period of life when the beneficiary is supposed to have learned how to invest his money, or mayhap, when he needs it to live upon, is preferable in every way. This brings the burden of meeting premiums in the productive time of life when

* Extract from address before the *National Dental Association*, Louisville, January 26, 1917, and reprinted from *Journal of N. D. A.*

it can best be borne, and the maturity late in life when it is most needed. Meanwhile, your family is protected against want.

When it comes to the investment of money for profit, in other words investing your money so that it will make more money for you, it requires the very greatest care not to make mistakes. No plan is infallible, and no system absolutely safe, but there are several forms of investment which seem to your essayist to be more suited to professional men than any others. First mortgages on approved real estate is one, old standard dividend—paying stocks is another, and bonds another. As for mortgages, they must be selected with care on property the value of which you have certain knowledge, either at first hand or through some real estate appraiser of repute.

Stocks should be dealt in conservatively and cautiously—always as an investment and not a speculation. By that is meant that the professional man should not buy or sell stocks on a margin. Aside from the constant risk of loss there is the inevitable diversion of interest from professional duties. No dentist can do good work at the chair while his mind is on the stock-ticker, and few dentists can afford to either win or lose in this kind of a game. The dentist's money is too hard earned to lose in such a manner, and the psychology of the situation is such that it is dangerous for him to win. If a man who earns his money as slowly and laboriously as does a dentist suddenly makes money by speculation, it is not within the power of human nature to convince him that he can not make more the same way. He takes "flyer" after "flyer" till the inevitable result is that he quits a loser instead of a winner. Many a professional man has wrecked his future in this way. If stocks are bought they should be paid for outright and the certificates placed in the safe or safety deposit vault; then if there should be a slight fluctuation the buyer is not disturbed by it. There is no objection to a dentist making money in stocks by buying when they are low and selling when they are high, and this can frequently be done by keeping one's head and not being too anxious to make large profits. In a general way the time to buy is when everybody else has gone mad and sold in a panic on the theory that the whole country has gone to destruction. There is invariably a period of stagnation after such an event when the observant buyer can pick up some stable stocks at a very reasonable price. Conversely the time to sell, if you sell at all, is when the frenzy of speculation has carried all stocks beyond their actual value, and the public seem to proceed on the theory that there is no limit to the advance. Remember that in stocks as with everything else there is a sane level to be reached some day, and the man who has not compromised himself by buying on a margin can afford to wait.

But after all, in the opinion of your essayist, the very best investment for a dentist is bonds. This sounds like tame advice. In truth there is little excitement about dealing in bonds, neither are there sudden or enormous profits. But for safety and convenience, and for steady and certain income there is nothing to compare with bonds. And in the long run what seems to start out slowly will after a few years begin to accumulate very rapidly.

Let it always be understood that as interest on mortgages, dividends on stocks, or coupons on bonds mature and come into your possession, you should no more think of using them for current expenses than if they did not belong to you. They must always be looked upon as a fund entirely separate from an expense fund. You paid your expenses without them before, and you can do it now. These profits on your investments should invariably be reinvested so that they in turn begin to make more money for you. It is in this way that bonds eventually turn out after all a very rapid way of making money, and the accumulations in a few years begin to mount up in a most surprising manner.

